# Polymethylenetetrazole: Synthesis, Characterization, and Energetic Properties

**DOI:** 10.3390/molecules29143389

**Published:** 2024-07-18

**Authors:** Ljubica Brenjo, Aleksandar Oklješa, Matija Tomšič, Berta Barta Holló, Jovica Nešić, Elvira Tóth, Črtomir Podlipnik

**Affiliations:** 1 Faculty of Sciences, University of Novi Sad, Trg Dositeja Obradovića 3, 21000 Novi Sad, Serbia; aleksandar.okljesa@dh.uns.ac.rs (A.O.); berta.hollo@dh.uns.ac.rs (B.B.H.); elvira.djurdjic@df.uns.ac.rs (E.T.); 2 Faculty of Chemistry and Chemical Technology, University of Ljubljana, Večna pot 113, 1000 Ljubljana, Slovenia; matija.tomsic@fkkt.uni-lj.si; 3 Military Technical Institute (VTI), Ratka Resanovića 1, 11132 Belgrade, Serbia; jovica.nesic@beotel.net

**Keywords:** tetrazoles, polymers, high-energy density materials

## Abstract

The tetrazole moiety remains one of the most interesting scaffolds in the development of new high-energy density materials (HEDMs) because of its desired characteristics, such as high nitrogen content and heat of formation (HOF). The combination of several heterocycles with high HOF seems to be a promising strategy for obtaining energetic materials with superior properties. Herein, we report the synthesis and characterization of a tetrazole polymer, polymethylenetetrazole (PMT), as a potential HEDM. The compound was characterized using NMR, IR, and Raman spectroscopy. Its weight average molecular mass was obtained by static light scattering (SLS), and its physical properties by powder XRD analysis. The density, sensitivity to friction (FS), and impact (IS) of the compound were determined as well. The results of the thermal and energetic properties of PMT suggest that this polymer could be an insensitive explosive.

## 1. Introduction

Energetic materials (EMs) include propellants, explosives, and pyrotechnics. They have a variety of military and industrial applications. The need for EMs with higher safety and energy content and lower cost of production is constantly increasing [1]. Some other desired characteristics of new high-energy-density materials (HEDMs) are high density, positive heat of formation (HOF), positive oxygen balance (OB), high detonation velocity and pressure, high thermal stability, simple synthesis, low sensitivity toward impact and friction, and environmental compatibility [2].

The traditional organo-nitro explosives TNT, RDX, and HMX derive their energy from the oxidation of the carbon backbone, whereas hepta- and octanitrocubanes possess very high densities and cage strains which enhance their energy. The third class of explosives derives most of their energy from their very high positive HOF. One of the main drawbacks of organo-nitro explosives is their toxicity and environmental activity [3]. 

Tetrazoles are an important constituent of energetic materials because of their unique properties: (1) having high nitrogen content and ring strain, tetrazole compounds may possess high density and HOF, and release significant quantities of energy and gases upon decomposition and explosion; (2) because of their aromaticity, tetrazole rings are thermodynamically stable in acidic and basic conditions, as well as in conditions of prolonged heating and boiling; (3) the explosive properties of tetrazole derivatives can be modified by the addition or removal of various functional groups; and (4) tetrazoles are more environmentally friendly than traditional organo-nitro explosives, as their decomposition products predominantly consist of nitrogen gas [4].

Within new explosives based on the tetrazole moiety, the tetrazole ring can be found in its neutral form containing energetic groups with oxygen, such as nitro groups and nitramines [5,6], and non-oxygen energetic groups such as the azide group [7]. In other explosive materials, the tetrazole ring is found in its anionic state, as a tetrazolate, where the positively charged component of tetrazolate salts includes metal ions [8] or organic nitrogen-rich cations such as ammonium, hydroxylammonium, guanidinium, and mono-, di-, and triaminoguanidinum ions [9]. On the other hand, tetrazolium salts were also developed as potential HEDMs [10], as well as salts that combine the tetrazolium cation with a tetrazolate anion in the same compound [11]. Energetic materials also include metal complexes (Co^3+^, Cd^2+^, or Fe^2+^) with the tetrazole moiety as a ligand [12,13,14]. Last but not least, the tetrazole ring is combined with other heterocyclic compounds with high nitrogen content, such as tetrazine [15], or with heterocycles that also have a high heat of formation, such as furazan, to obtain single molecules with higher HOF [16].

Obtaining new tetrazole compounds as potential HEDMs with improved properties is still a current topic of research. Singh et al. reported facile syntheses of ethane and ethene-linked bistetrazole derivatives and their dihydroxylammonium salts [17], where one compound was shown to have superior energetic properties than RDX. Another example is compounds where the tetrazole moiety was tethered directly to 1,3,4-oxadiazole, as a more stable oxadiazole isomer than furazan [18], which contributed to better chemical and thermal stabilities of the compounds. Aside from the examples where the tetrazole ring is connected to another azole ring directly or by a linker, worth mentioning are fused heterocycles, such as pyrazolo[1,5-*d*]tetrazole-based energetic salts [19]. These compounds were found to be promising candidates for application as secondary and primary explosives.

A recent review covers high-performance energetic materials obtained by combining the tetrazole ring with 1,2,4-oxadiazole [20]. Additionally, another review summarizes the synthesis and energetic characteristics of tetracyclic tetrazole, furazan-tetrazole, 1,2,4-triazole-tetrazole, and pyrazole-tetrazole derivatives, with the underlying idea that the combination of several heterocyclic rings is an efficient strategy to construct novel energetic materials with superior performance [21].

This could mean that a chain consisting of tetrazole moieties bound with each other may have very high HOF and nitrogen content. During the optimization of the synthesis of 5-hydroxymethyl-tetrazole from 5-chloromethyl-tetrazole, we observed from NMR spectra that a small portion of the starting material was converted to a polymer structure in alkaline conditions. That inspired us to synthesize a polymer from 5-chloromethyl-tetrazole, which could be used as a potential HEDM. Herein we report the synthesis and characterization of the obtained tetrazole polymer, polymethylenetetrazole (PMT) with IR, NMR, XRD, Raman, static light scattering (SLS), and TGA/DSC analyses. By the end of our research, we discovered a paper that describes the synthesis of PMT [22]. However, the authors reported different reaction conditions in obtaining the polymer. Furthermore, they described a rather oligomeric nature of the reaction products, with 15 to 20 repeating units. The focus of the paper is shifted more towards branched tetrazole-containing polymers, so the information about their linear tetrazole is scarce. From the results we obtained, it is safe to assume that we obtained a long-chained tetrazole polymer with the potential of being a HEDM.

## 2. Results and Discussion

### 2.1. Synthesis and Analysis of NMR and IR Spectra

**Scheme 1:**
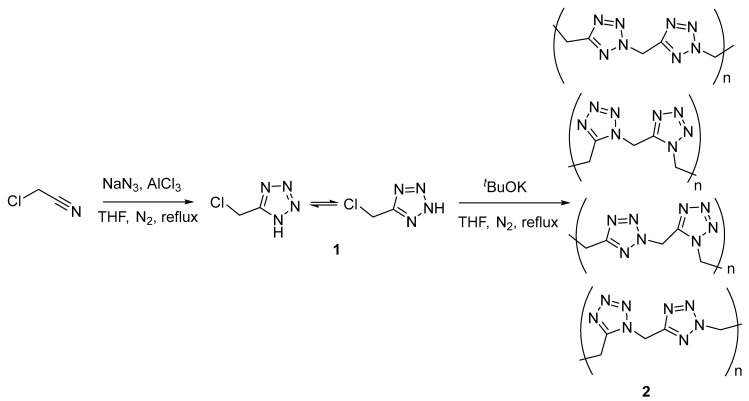
The two-step synthesis of polymer **2**.

The synthesis of polymer **2** was achieved by mixing 5-chloromethyl-tetrazole (**1**) with a strong sterically hindered base such as potassium *tert*-butoxide (Scheme 1). We suggest that in this way the deprotonation of **1** is favored over the nucleophilic substitution of the chloride in **1** by the bulky *tert*-butoxide anion. After the deprotonation of a single molecule of **1**, we obtained the matching tetrazolate anion. The anion contains two distinct types of nitrogen atoms. The first type, adjacent to the tetrazole carbon, will be named N1, and the second type, which consists of two nitrogen atoms more distant from the carbon atom, will be named N2. As the tetrazolate approaches another molecule of **1**, it acts as a nucleophile and substitutes the chloride from **1**, which results in a dimer. Such a dimer can also lose its proton, and the chain reaction propagates in building polymer **2**. As can be seen in Scheme 1, we expect to see four different configurations within a single chain, or in different polymer chains as a result of two types of nitrogen involved in nucleophilic attack.

We repeated the reaction with different times that varied from 2 to 4, 8, 24, and 48 h. The mass of the product was ascending with the reaction times, and the smallest mass difference was between 24 and 48 h, which convinced us that 48 h is enough to obtain the best possible yield. Also, it is important to ensure that the THF does not evaporate too much over time, and it is best to use a pear-shaped flask and controlled heating on the boiling point of THF for reactions that last longer than 4 h. The procedure above was repeated four more times, and we obtained 0.2447 g, 0.2934 g, 0.3010 g, and 0.2326 g of compound **2**. So the average mass would be 0.2672 g with a standard deviation of around 30 mg.

For the NMR spectra of the polymer, DMSO-d6 was used as a solvent, but the polymer was only partially dissolved. Some bigger particles of the powder would not go into the solution, so the suspension was filtered and the clear solution was transferred to the NMR tube. The spectra were very similar, independent of the reaction times in terms of chemical shift, so only the spectra of the polymer after 48 h will be discussed below.

**Figure 1:**
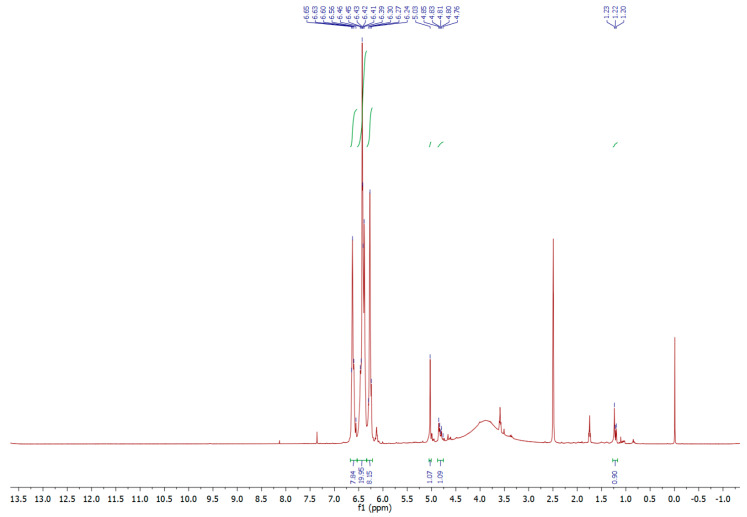
^1^H NMR spectrum of compound **2**.

In the ^1^H NMR spectrum of compound **2** shown in Figure 1, the most intense signals were in the range of 6.24 to 6.65 ppm and they may correspond to the protons of the methylene groups. We can observe a large downfield shift from 4.95 ppm in the monomer molecule to signals above 6 ppm, which was expected because each methylene group is connected to two electron-withdrawing tetrazole moieties. Less prominent signals in the range of 4.76 ppm and 5.03 ppm were observed as well. What can be observed, aside from the signals of methylene protons, are the signals at 1.75 ppm and 3.59 ppm which stem from THF. As described in the synthesis section, the polymer was vacuum dried, but this did not change the mass of the polymer. We thus explain the presence of THF not as a consequence of insufficient washing and drying, but of the trapping of THF molecules during the formation of the bulky polymer molecules. THF is liberated once the polymer is dissolved in DMSO-d6, and detected in the spectra.

**Figure 2:**
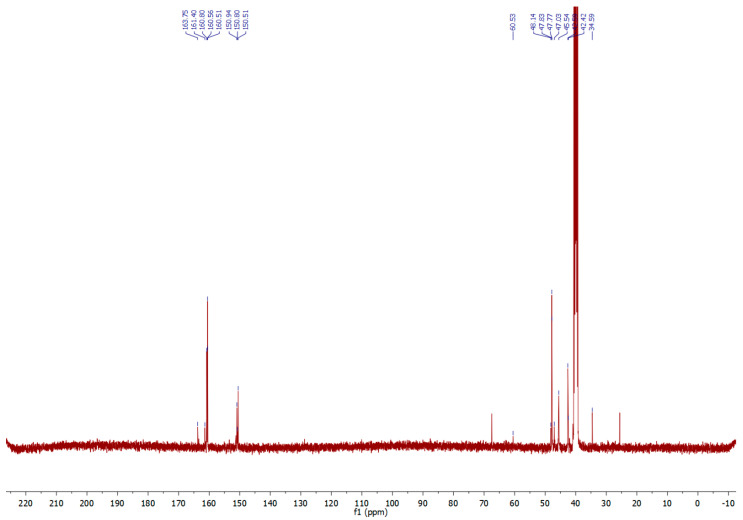
^13^C NMR spectrum of compound **2**.

What can be observed in the ^13^C NMR spectrum of compound **2** shown in Figure 2 are three groups of signals: around 150 ppm, around 160 ppm, and in the range of 34.6–60.5 ppm. The last group of signals corresponds to the methylene carbon atoms. The group of signals around 160 ppm matches the carbon atoms in 2,5-substituted tetrazole moieties, and the signals around 150 ppm fit the carbon atoms in 1,5-substituted tetrazoles. The signals at 25.59 ppm and 67.49 ppm correspond to THF but shifted downfield by 0.45 ppm and 0.46 ppm, respectively, in comparison to the literature values [23]. From the spectrum, one could argue that the 2,5-substituted tetrazole moieties are more frequent in the polymer coils than the 1,5-tetrazole monomers. It can be seen that there are five signals in the 160 ppm region against three signals detected in the 150 ppm region, and not only the number of signals is higher in the first case, but also the signals are more intensive. It is worth noting that in the work of Kizhnyaev et al. [22], only two signals in the ^13^C NMR spectrum were mentioned, one corresponding to the 1,5-tetrazole moiety, and the other corresponding to the 2,5-tetrazole. As we deal here with long polymer chains, the many different chemical shifts can be rationalized not only with the differing configuration of the 1,5- and 2,5-tetrazole units but with the conformations that the polymer coils can have in a solution.

**Figure 3:**
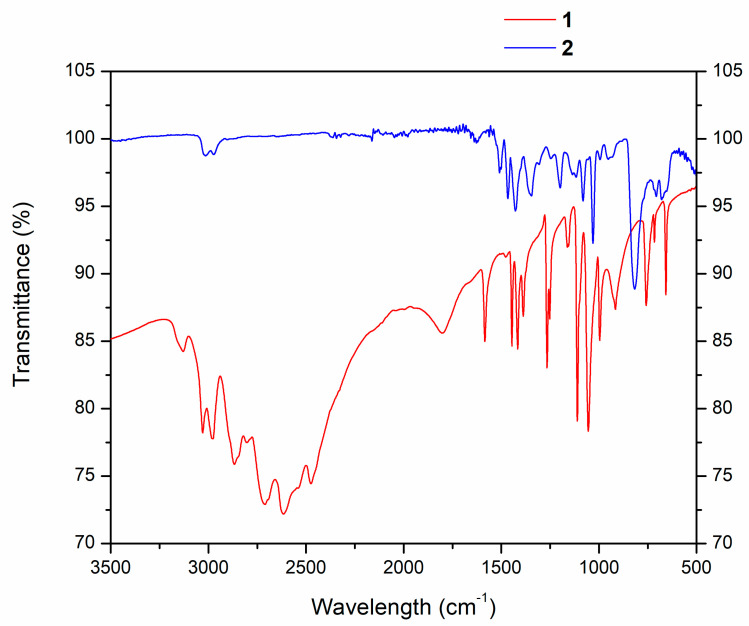
IR spectra of compound **1** (red) and compound **2** (blue).

Comparing the IR spectra of the starting compound **1** shown in Figure 3 (red) and the final polymer **2** shown in Figure 3 (blue), we can observe band absorptions at different frequencies and different band intensities. The most noticeable difference is in the region from 3100 to 2400 cm^−1^, where compound **1** has vibrations at 3029, 2976, 2867, 2710, and 2616 cm^−1^, whereas compound **2** shows only one vibration at 3014 cm^−1^. The broad absorption in the region of 2867–2616 cm^−1^ indicates the presence of NH···N hydrogen bonds, which are generally found in five-membered heteroatomic compounds with two or more nitrogen atoms in the ring [24]. In the region of 1600–950 cm^−1^ the band frequencies, intensities, and shapes are quite similar for both compounds, corresponding to the various C-N and N-N vibrations within the ring present in both molecules, comparable with some known 5-substituted tetrazoles [25]. The region from 900 to 400 cm^−1^ shows one major change, namely in the spectrum of polymer **2** we observe a strong absorption at 817 cm^−1^ not present in the spectrum of compound **1**.

### 2.2. XRD Analysis

**Figure 4:**
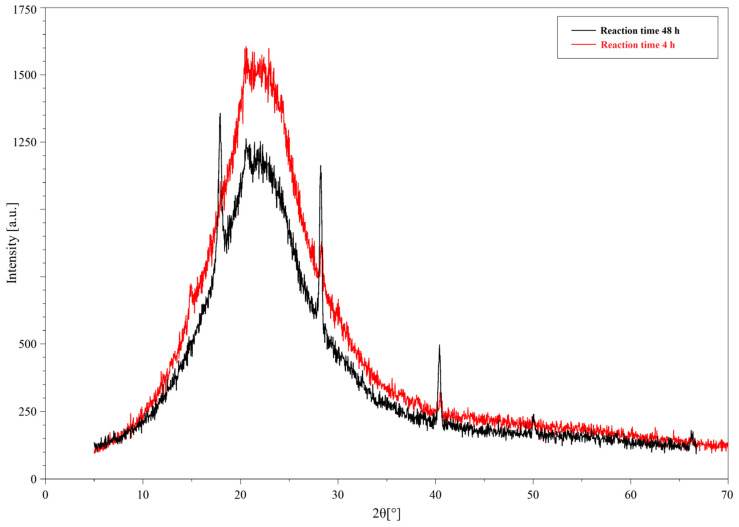
XRD spectrum of compound **2**.

The results of the XRD analysis are shown in Figure 4. The experiments were completed on compound **2**, which was obtained after the reaction time of 4 h (red graph) and 48 h (black graph). We can observe that the polymer shows an overall amorph structure, with four crystal centers present in the graph corresponding to the polymer obtained after 4 h, and five crystal centers observed for the polymer obtained after 48 h of reflux. Four of them, namely 28.13, 40.37, 50.06, and 66.2, correspond to potassium chloride, KCl, which is a side product of the reaction. Even though compound **2** was washed with distilled water several times, clearly some of the KCl was trapped in the polymer coils, similar to the trapping of THF molecules described above. Furthermore, the concentration of KCl was higher in the case of prolonged reaction time, which can be deduced from the higher spikes in the black graph. This occurrence may be explained by the longer chains that are formed over time, so more of the inorganic salt gets trapped in the polymer coils. The other explanation is that with prolonged reaction times, the polymer has more time to organize itself around the potassium ion. The only crystal center not corresponding to KCl, observed only for the polymer obtained after 48 h of reflux, is at 17.87°. We think that the tetrazole moieties in the polymer coil can form some sort of ordered structures around KCl units, which are prevalent at higher KCl concentrations. An interesting fact that was observed during the analysis is that the polymer underwent a series of small explosions, which may suggest that the wavelength used was enough to initiate a chemical reaction.

### 2.3. Molecular Weight Determination

**Figure 5:**
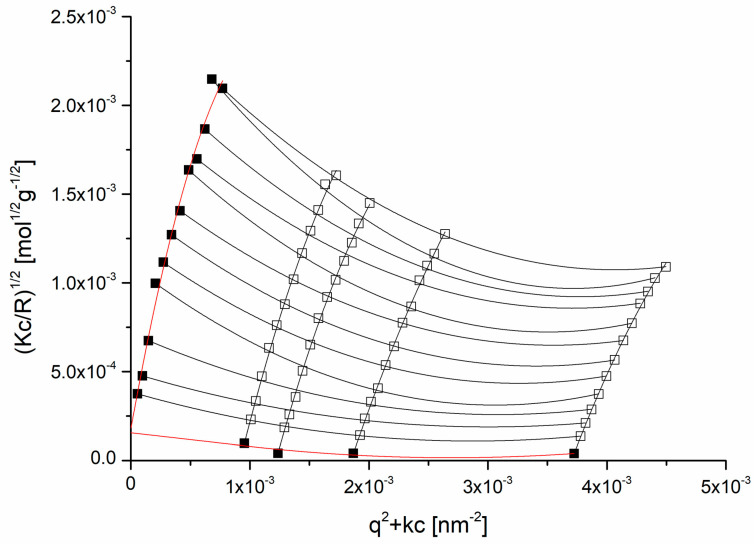
SLS results for the solutions of compound **2** from 0.0190 mg/mL to 0.0745 mg/mL in the form of a Berry plot. The extrapolations to zero polymer concentration and zero scattering angle are marked with filled squares.

Based on the Berry plot analysis of light scattering at 638 nm shown in Figure 5, the weight average molecular weight (Mw) of compound **2** was determined to be around 3.22·10^7^ g/mol, and the obtained value of the second virial coefficient was −1.04·10^−3^ mLmol/g^2^. The radius of gyration (Rg) was about 357 nm. The difference between the Berry plot and the more known Zimm plot is in the quantity used on the ordinate—the Berry plot takes the square root of Kc/R_θ_ values, whereas the Zimm plot takes the Kc/R_θ_ values as such, where K is the optical constant, c is the mass concentration of the solutions, and R_θ_ is the excess Rayleigh ratio. For the analysis of particles with a larger radius of gyration than 50 nm, the Berry method is superior to the Zimm method in terms of accuracy and robustness [26], which was the case in our analysis. The negative second virial coefficient suggests that particle–particle attractive interactions are favored in the system and that particle aggregation is likely to occur [27]. The Mw value seems to be quite large, which could be an indication of possible polymer aggregation, which could be related to the observed very low solubility of the polymer. Namely, when performing the SLS experiments and making efforts to determine the Mw, we had to cope with the problem of the generally very low solubility of the polymer, and eventually had to be satisfied with the selection of the solvent that seemed to dissolve the polymer best.

### 2.4. Thermal Properties of ***1*** and ***2***

**Figure 6:**
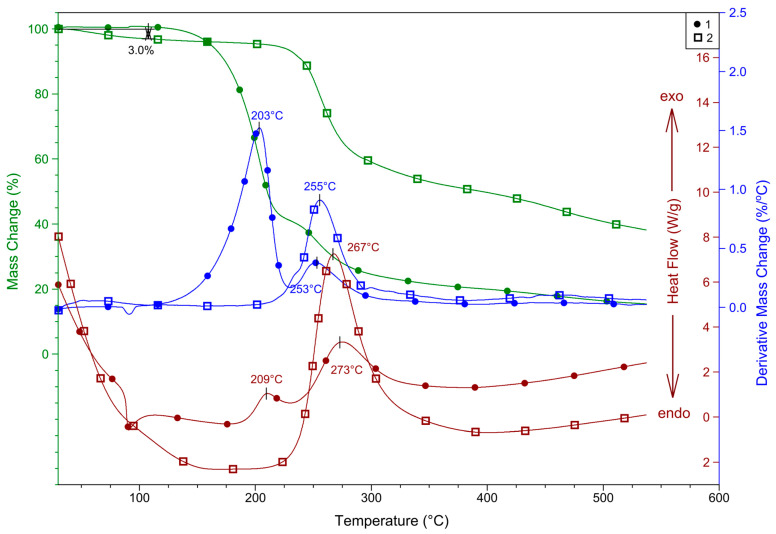
TG, DTG, and DSC curves of compounds **1** and **2** in argon.

The tetrazole derivatives were analyzed in the atmosphere of argon to avoid their potentially intense decomposition. The 5-chloromethyl-1*H*-tetrazole (**1**) melts at 87 °C (Figure 6), but its thermal decomposition starts at 135 °C, onset. Above this temperature, compound **1** loses 59.6% of its mass. The corresponding DTG maximum appears at 203 °C. This step partially overlaps with the next one, which appears at 253 °C. In this step, the sample loses an additional 17.7% of mass. Above 300 °C, the decomposition continues at a steady rate and does not finish up to 550 °C. Without further analysis of the evolved gases, it is not possible to determine the decomposition mechanism of compound **1**. Simultaneously with thermogravimetric (TG), differential scanning calorimetric (DSC) analysis was also conducted. After the melting of compound **1**, the very beginning of the decomposition is followed by a light endothermic effect, which turns into exothermic above 180 °C. Despite the higher mass loss in the first decomposition step, its exothermicity is significantly lower than that of the second decomposition step with lower mass loss. It suggests that the part of compound **1** with higher energy density decomposes at higher temperatures.

The thermal properties of polymethylenetetrazole (**2**) obtained by polymerization of **1** are different. Compound **2** begins to lose mass at room temperature. Considering that this mass loss step is the most intense up to ~100 °C, it is most probably caused by the evaporation of the adsorbed solvent. However, to prove the nature of the solvents, additional evolved gas analysis measurements are necessary. The desolvated compound **2** is more stable than compound **1** and begins to decompose at an onset temperature above 211 °C. Above 300 °C, the decomposition continues at a steadier rate and does not finish up to 550 °C. The TG and DTG curves suggest that the most intense decomposition step occurs in several overlapped processes that cannot be distinguished. During these processes, compound **2** loses 35.7% of its mass. At higher temperatures in the steadier phase of the decomposition, the additional mass loss is 20.8%. The DSC curve shows an endothermic effect from room temperature to ~160 °C, following the supposed evaporation, and a significant exothermic effect with a peak maximum at 267 °C during the decomposition of compound **2**. When the reaction time to obtain polymeric compound **2** is longer, e.g., 48 h, the product shows the same characteristics as described above up to ~240 °C. Above this temperature, the sample explodes. During the heating of the compounds in the air, they exploded also.

### 2.5. Energetic Properties of PMT

Density is an important parameter for the prediction of combustion and detonation parameters of novel energetic materials. The average density and standard deviation were estimated from the results of 15 experiments (Table S1). The experimental density of polymethylenetetrazole (PMT) is 1.557 g/cm^3^. This kind of linear tetrazole polymer where the tetrazole units are connected directly by methylene groups demonstrates an enhanced density compared to polymers where the tetrazole units are connected by a methylene group to the main glycidyl chain, as in p-GAT that has a density of 1.436 g/cm^3^ [28]. The standard deviation in estimating the density of the polymer under study was not above 0.0057 g/cm^3^.

The determination of the sensitivity towards friction (FS) and impact (IS) of compound PMT gave a friction sensitivity > 360 N and an impact sensitivity > 100 J. As a result, the PMT is insensitive to friction and impact.

## 3. Materials and Methods

### 3.1. Materials and Measurements

All reagents and solvents were obtained from commercial sources and used without further purification unless stated otherwise.

All ^1^H NMR, ^13^C NMR, and ^1^H-^15^N HMBC NMR spectra were obtained using a Bruker AVANCE III 400 (Bruker, Rheinstetten, Germany) spectrometer operating at 400 MHz (^1^H), 100.6 MHz (^13^C), and 40.6 MHz (^15^N), and residual solvent signals were used for the chemical shift calibration for ^1^H and ^13^C NMR spectra (ppm; δ scale). ^15^N Chemical shifts were measured relative to an external sample of nitromethane (nitromethane set as 380.2 ppm). For the ^1^H NMR spectra, multiplicities are given as s (singlet) and om (overlapping multiplets).

Melting points were determined using a Stuart Melting Point SMP10 apparatus (Cole-Palmer Ltd., Staffordshire, UK), and are uncorrected.

IR spectra were recorded using a PerkinElmer Spectrum Two (PerkinElmer, Seer Green, UK) FT-IR spectrometer (wavenumbers in cm^−1^) and using an FT-IR spectrometer Nicolet iS20 (Thermo Scientific, Madison, USA) equipped with an ATR accessory.

XRD analysis was completed using a Rigaku MiniFlex600 diffractometer (Rigaku Corporation, Tokyo, Japan) using Ni-filtered CuKα radiation. Further examination of the samples was completed by Raman spectroscopy at room temperature using a Thermo Fisher Scientific DXR™ Raman Microscope (Thermo Fisher Scientific Inc., Madison, WI, USA). The device is equipped with a DPSS (Diode Pumped Solid State) laser using λ = 780 nm excitation and coupled with a CCD camera as a detector.

The molecular mass of the polymer was elucidated by static light scattering (SLS) obtained using a 3D-DLS Spectrometer (LS Instruments, Fribourg, Switzerland), equipped with a 35 mW He-Ne laser (λ = 632.8 nm), high-precision beam-splitter, and two single-mode fiber-optic detectors with avalanche photodiodes (photodetection efficiency > 65%). Samples in quartz cylindrical cuvettes (Hellma; path length of 8 mm) were immersed in a large-diameter thermostated bath (25 °C) of index-matching liquid (decalin). A number of 30 s DLS measurements were performed in the 3D-cross-correlation scheme within the range of scattering angles from 30° to 150° with a step of 10° (excluding the angle of 140°) and at least 10 of them were averaged to obtain the final result—the corresponding average scattering intensity was used as the SLS data. The toluene was also measured as the reference sample. SLS intensities of the measured samples were put on an absolute scale by calculating the Rayleigh ratio Δ*R*(*θ*):

$$\Delta R\left(\theta \right)=\frac{\langle I_{S}\left(\theta \right)\rangle -\langle I_{0}\left(\theta \right)\rangle }{\langle I_{r}\left(\theta \right)\rangle }\cdot \left(\frac{n_{sol}}{n_{r}}\right)\cdot R_{r}\left(\theta \right),$$

where 〈*I_s_*(*θ*)〉, 〈*I*_0_(*θ*)〉, and 〈*I_r_*(*θ*)〉 represent the scattering intensity of the sample, solvent, and reference, respectively. *R_r_*(θ) stands for the Rayleigh ratio of the reference, *n_r_* for the refractive index of the reference, and *n_sol_* for the refractive index of the samples.

The densities of the polymer solutions were measured at 25 °C utilizing the Anton Paar density meter DSA 5000M. The refractive index increment dn/dc was determined for the polymer in a formamide-water (90:10, *v*/*v*) mixture with the differential refractometer DnDc-2010 WGE DR. BURES.

Thermal data were collected using a TA Instruments SDT Q600 thermal analyzer. The decomposition was followed from room temperature to 550 °C at a 20 °C/min heating rate in the argon carrier gas (flow rate = 100 cm^3^/min). Sample holder/reference: alumina crucible/empty alumina crucible, and sample mass was 1.5–2 mg.

The densities of the synthesized PMT samples were experimentally measured on a gas pycnometer Ultrapyc 5000 (Anton Paar, Graz, Austria). The experiments were performed with an inert gas supply of nitrogen. PMT was placed in a sample cell in powder form. The temperature was set at 20.0 ± 0.8 °C.

Impact and friction sensitivity were determined according to standard methods using a Julius Peters, Berlin NW 21 apparatus (Julius Peters GmbH, Berlin, Germany).

Impact and friction sensitivities were determined according to STANAG standards [29,30] on BAM-type machines. The full series of experiments includes 25–30 trials with the powdered sample loaded with 40 mm^3^ (for impact) and 10 mm^3^ (for friction tests) spoons. The particle size of the samples was not controlled, and synthesized powders were mostly finer than that specified by STANAG 4489 [29] (0.5–1 mm), but conform to STANAG 4487 [30] (less than 0.5 mm).

The impact sensitivity of PMT was examined experimentally according to standard methods using a Julius Peters Impact Machine (Julius Peters GmbH, Berlin, Germany) in accordance with Annex C in STANAG 4489 [29]. The results are reported in terms of 50% probability of decomposition. The friction sensitivity of PMT was measured with a Julius Peters Friction Machine (Julius Peters GmbH, Berlin, Germany) in accordance with Annex A in STANAG 4487 [30].

### 3.2. Synthesis

The synthesis of the polymethylenetetrazole (PMT) was completed in two steps (Scheme 1). Firstly, precursor **1** was synthesized from chloroacetonitrile (1 eq.), aluminum-chloride (1 eq.), and sodium-azide (3 eq.) in THF under reflux and an inert atmosphere, following a slightly modified procedure from the literature [31,32]. Then, compound **1** was heated under reflux with one equivalent of potassium *tert*-butoxide in THF under a nitrogen atmosphere, which yielded our polymer **2** as a white precipitate.

#### 3.2.1. 5-Chloromethyl-1H-tetrazole (**1**)

In a previously dried and nitrogen-filled round bottom flask, 6.16 g (0.09475 mol) of sodium-azide, 2 mL (0.03160 mol) of chloroacetonitrile, and 20 mL of freshly prepared absolute tetrahydrofuran (THF) were added and the suspension was stirred. In the meantime, in a separate beaker, 4.22 g (0.03165 mol) of dry aluminum chloride was dissolved in 40 mL of abs. THF. The AlCl_3_ solution was added to the flask, then the beaker was washed once with 10 mL of abs. THF and this was also added to the flask. The system was closed and purged with nitrogen gas for 5 min, and then the reaction mixture was heated under reflux for 25 h. The reaction mixture obtained over time a slightly brown color. After cooling to room temperature, 34 mL of diluted hydrochloric acid, HCl 1:1, was added to the reaction mixture, and the suspension was filtered. The clear yellow filtrate was transferred to a separation funnel and extracted with 30 mL of methylene-chloride, CH_2_Cl_2_. The organic phase was separated from the water phase, dried with anhydrous sodium-sulfate, Na_2_SO_4_, and concentrated in vacuo, which yielded a light yellow solid (3.4553 g). The crude product was recrystallized with 5 mL of ethylene-chloride, giving 2.22 g (59%) of colorless crystals.

Compound **1**: m. p. 88 °C. ^1^H NMR (400 MHz, D_2_O, TMS): δ = 4.95 (s, 2H). ^13^C NMR (100.6 MHz, D_2_O, TMS): δ = 31.8 and 154.6. IR: 3029 (m), 2976 (m), 2867 (m), 2710 (s), 2616 (s), 1804 (m), 1584 (m), 1446 (m), 1416 (m), 1388 (m), 1266 (m), 1253 (m), 1162 (w), 1111 (s), 1054 (s), 996 (m), 916 (m), 758 (m), 716 (w), and 657 (m) cm^−1^.

#### 3.2.2. Polymethylenetetrazole (**2**)

Reaction time 4 h: In a dried and nitrogen-filled flask, compound **1** (0.5003 g; 0.0042 mol) and potassium *tert*-butoxide (0.4736 g; 0.0042 mol) were added, followed by adding 10 mL of absolute THF. The system was closed and nitrogen was brought into the mixing reaction mixture for 5 min. The mixture was brought to reflux. After 2 h, the formation of a white precipitate on the walls of the flask was already visible. The reflux was maintained for 4 h. After the reaction mixture cooled down to room temperature, distilled water was added, and the pH was adjusted to around 3 with HCl 1:1. Vacuum filtration gave a white solid which was washed several times with distilled water, and left to dry in the air for one to two days, and the final mass of the powder was 0.1827 g.

Reaction time 48 h: In a dried and nitrogen-filled flask, compound **1** (0.4999 g; 0.0042 mol) and potassium *tert*-butoxide (0.4735 g; 0.0042 mol) were added, followed by 10 mL of absolute THF. The system was closed and nitrogen was brought into the mixing reaction mixture for 5 min. The flask was immersed in an oil bath and the temperature was brought to 66 °C. After 2 h the formation of a white precipitate on the walls of the flask was visible. The stirring was turned up from 600 rpm to 750 rpm after 24 h, and the reflux was maintained for 48 h. After the reaction mixture cooled down to room temperature, and the majority of the solid phase settled down, the THF phase was transferred to a round bottom flask and concentrated in vacuo, which left some kind of colorless oil. In both flasks distilled water was added, and the pH was adjusted to around 3 with HCl 1:1. Vacuum filtration of both suspensions gave a white solid which was washed several times with distilled water, and left to dry in the air for one to two days, and the final mass of the powder was 0.2642 g. The product was additionally dried under vacuum, but the mass did not change.

^1^H NMR (400 MHz, DMSO-d6, TMS): δ = 1.20–1.23 (om); 4.76–4.85 (om); 5.03 (om); 6.24–6.30 (om); 6.39–6.46 (om); and 6.56–6.65 (om). ^13^C NMR (100.6 MHz, DMSO-d6, TMS): δ = 34.6; 42.4; 42.6; 45.5; 47.0; 47.8; 47.8; 48.1; 60.5; 150.5; 150.8; 150.9; 160.5; 160.6; 160.8; 161.4; and 163.8. IR (ATR): 3014 (w), 1509 (m), 1467 (m), 1428 (m), 1345 (m), 1198 (m), 1161 (w), 1081 (m), 1039 (m), 953 (w), 817 (s), 706 (m), 679 (m), and 481 (w) cm^−1^. Raman (780 nm): 665, 820, 1031, 1081, 1203, 1310, 1429, and 1509 cm^−1^.

### 3.3. Determination of Weight Average Molecular Weight with Static Light Scattering

The first step was to find an adequate solvent for dissolving the new polymer material. A variety of solvents of different polarity were tested at room temperature. The polymer is virtually insoluble in water, methanol, ethanol, 2-propanol, 1-nonanol, THF, ethyl-acetate, toluene, and chlorobenzene, and very slightly soluble in acetone. The polymer showed partial solubility in DMSO and DMF, but the best solubility was observed in formamide. To avoid reproducibility problems due to the high hygroscopicity of formamide, we used a mixture of formamide with distilled water in a volume ratio of 90:10 as a solvent. The density measurements of the solvent showed good repeatability, with a standard deviation of ±2·10^−6^ g/mL.

We tried to dissolve compound **2** after a reaction time of 48 h in the formamide-water mixture, but some bigger particles were still floating. Somewhat better solubility was observed for the powder obtained after drying the DMSO-d6 solution after NMR measurements, but still, the stock solution did not seem clear, so the light scattering results were highly questionable and are not included in this manuscript.

So we tried to dissolve the powder obtained after a reaction time of 4 h and the solubility was much better—we prepared a stock solution with a concentration of 0.14 mg/mL. The stock solution was diluted 2, 4, 6, and 8 times. The densities (ρ) were measured for all the solutions and the mass concentrations (c) were calculated from them. The ρ(c) function showed a clear linear dependence. Then, the dn/dc value was determined using the same solutions, at concentrations of 0.0190, 0.0247, 0.0374, 0.0745, and 0.1444 mg/mL, and the dn/dc value of (0.2158 ± 0.057) mL/g was obtained. The SLS experiments were performed for these solutions and the obtained results were reported earlier in the text.

## 4. Conclusions

The polymer polymethylenetetrazole (PMT) was synthesized in two steps. The compound was synthesized at different reaction times, where the highest mass was obtained with the longest heating period. The NMR and Raman spectra did not show any significant differences between polymers isolated after 4 h and 48 h of reflux. According to the static light scattering results, the obtained polymer has a high weight average molecular mass even when the reaction time is 4 h. With longer reaction times, PMT is less soluble. XRD analysis showed an amorph structure in both cases. Simultaneous TG-DSC analysis showed that the most intensive decomposition of PMT occurs above 190 °C, followed by a significant exothermic heat effect. With longer reaction times the decomposition rate of PMT increases and its explosive decomposition becomes more expressed. The polymer showed a high density of 1.557 g/cm^3^. The resistance to mechanical impacts makes this high-molecular tetrazole compound a promising candidate as a high-energy and insensitive explosive.

## Data Availability

The data presented in this study are available on request from the corresponding authors.

## Supplementary Materials

The following supporting information can be downloaded at: https://www.mdpi.com/article/10.3390/molecules29143389/s1, Figure S1: ^1^H NMR spectrum of compound **1**. Figure S2: ^13^C NMR spectrum of compound **1**. Figure S3: ^1^H NMR spectrum of compound **2** after a reaction time of 4 h. Figure S4: ^13^C NMR spectrum of compound **2** after a reaction time of 4 h. Figure S5: Raman spectra of compound **2** after reaction times of 4 h and 48 h. Table S1: Results of density measurements of PMT obtained by a nitrogen pycnometer. Figure S6: ^1^H-^15^N NMR spectrum of compound **2**. Figure S7: ^1^H-^15^N NMR spectrum of compound **2** (zoomed in).
