# Supplementary material for: Polymethylenetetrazole: Synthesis, Characterization, and Energetic Properties
Source: Molecules. 2024 Jul 18;29(14):3389. doi: 10.3390/molecules29143389 (PMC11280064; doi:10.3390/molecules29143389)
Supplement: Supplementary file 1 [file molecules-29-03389-s001.zip › molecules-3093910-supplementary.pdf]

Supplementary Materials

Polymethylenetetrazole: synthesis, characterization, and energetic properties

Ljubica Brenjo [1*], Aleksandar Oklješa [1], Matija Tomšič [2], Berta Barta Holló [1], Jovica Nešić [3], Elvira Toth [1], Črtomir Podlipnik [2*]

[1] Faculty of Sciences, University of Novi Sad, Trg Dositeja Obradovića 3, 21000 Novi Sad, Serbia
[2] Faculty of Chemistry and Chemical Technology, University of Ljubljana, Večna pot 113, 1000 Ljubljana, Slovenia
[3] Military Technical Institute (VTI), Ratka Resanovića 1, 11132 Belgrade, Republic of Serbia
* Correspondence: ljubica.brenjo@dh.uns.ac.rs; crtomir.podlipnik@fkkt.uni-lj.si

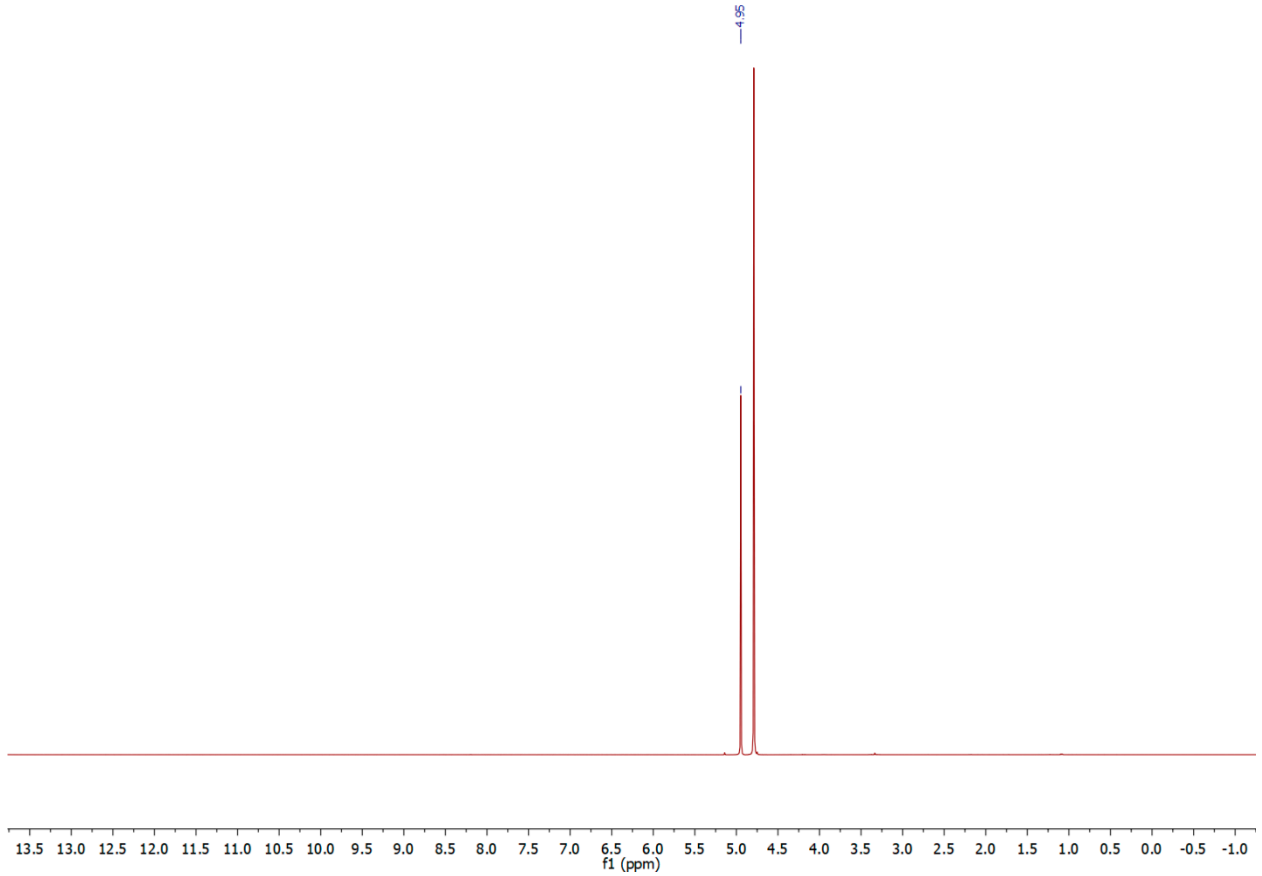

Figure S1. ^{1}H NMR spectrum of compound **1**.

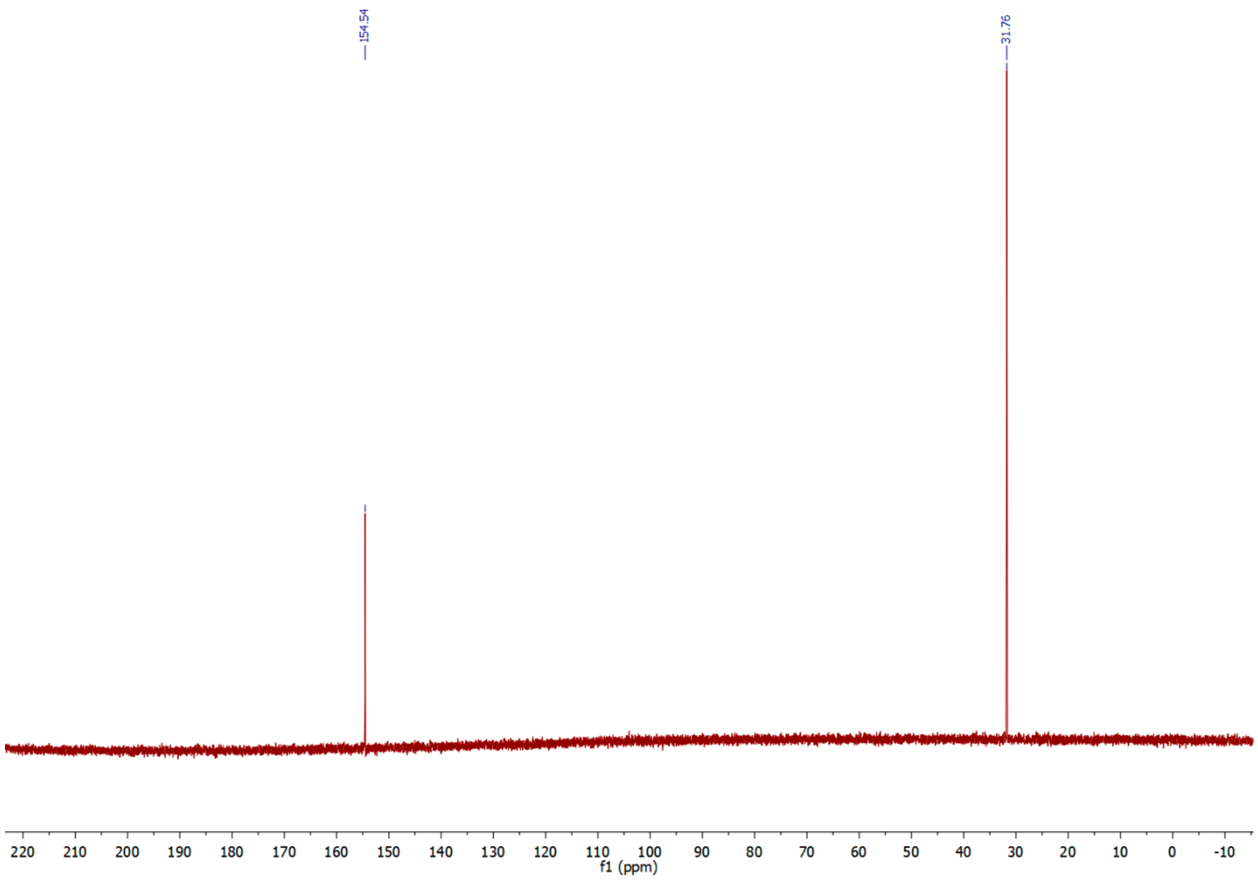

Figure S2. ^{13}C NMR spectrum of compound **1**.

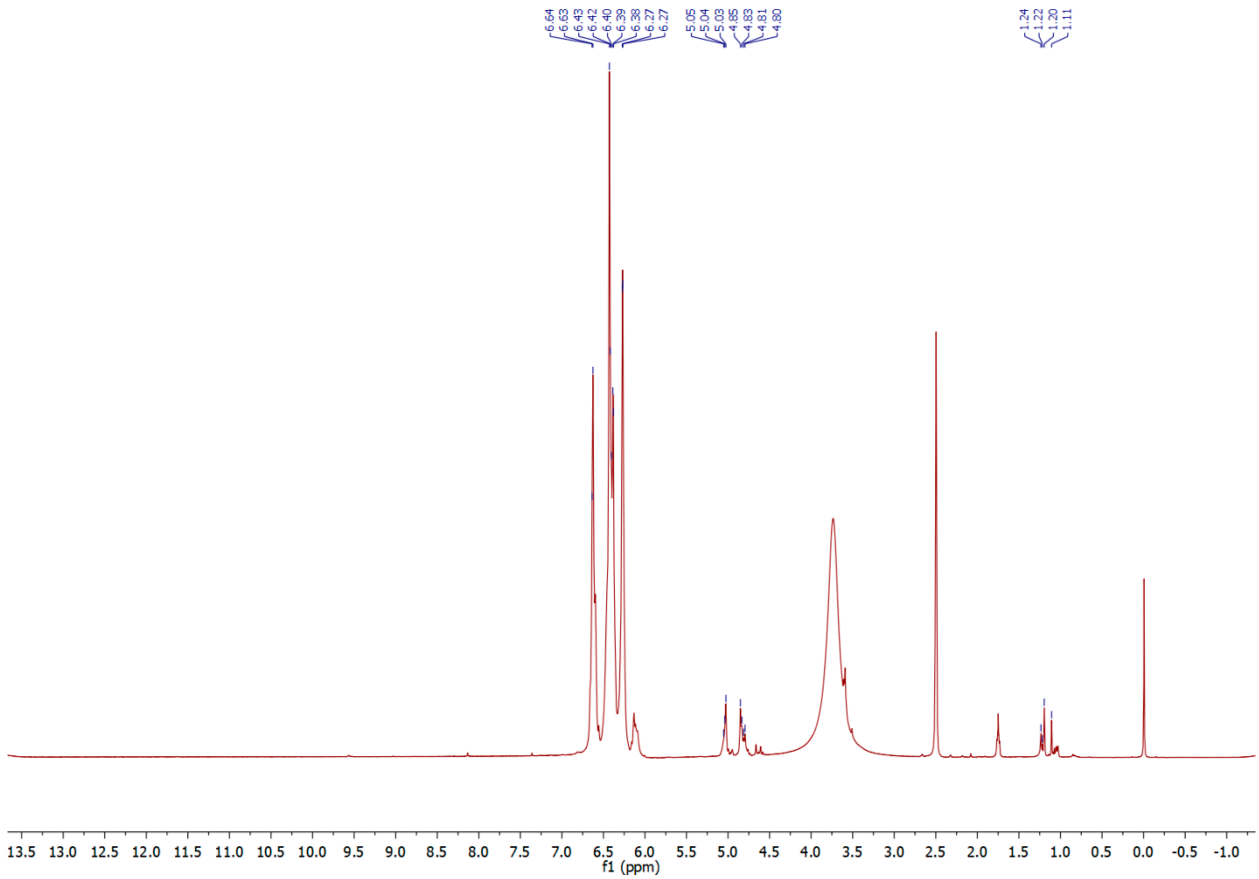

Figure S3. ^{1}H NMR spectrum of compound **2** after a reaction time of 4 h.

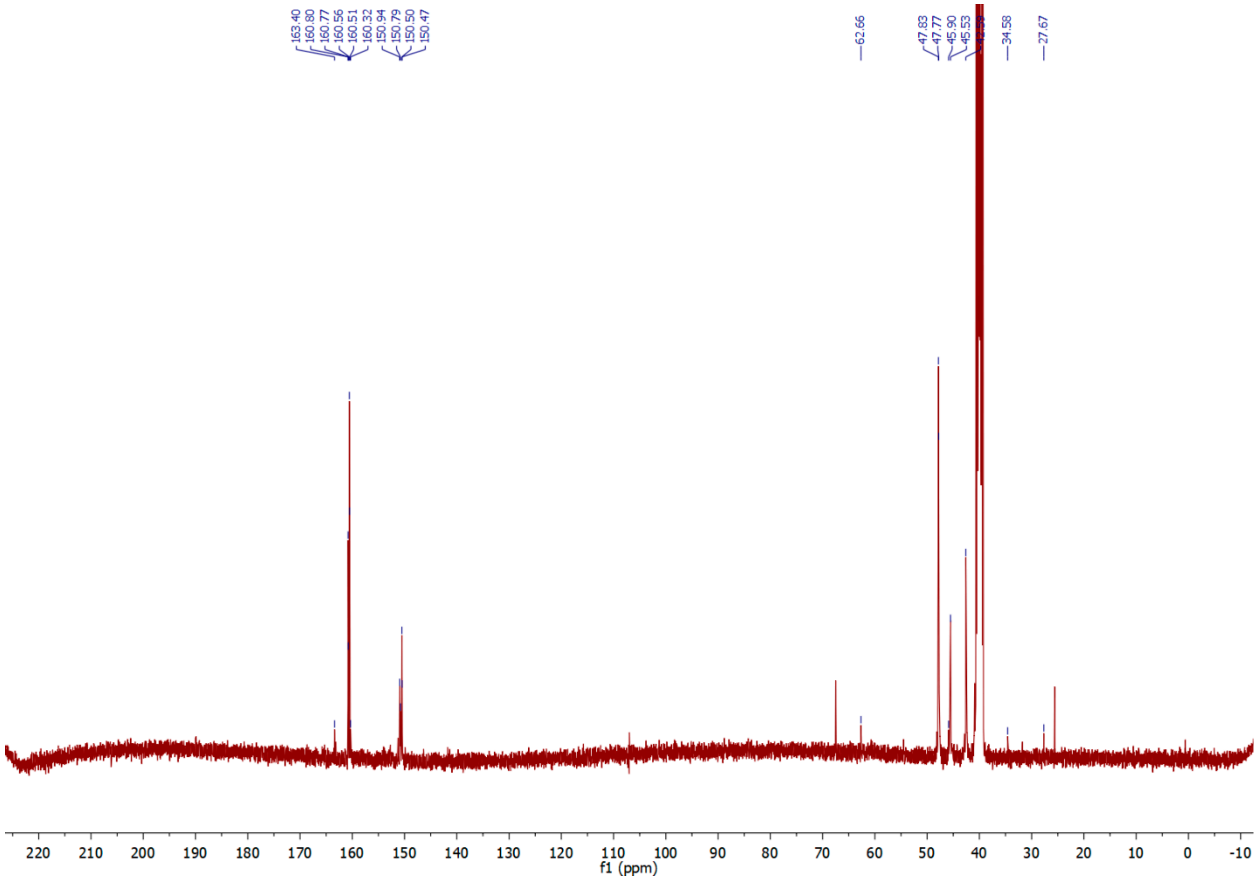

Figure S4. ^{13}C NMR spectrum of compound **2** after a reaction time of 4 h.

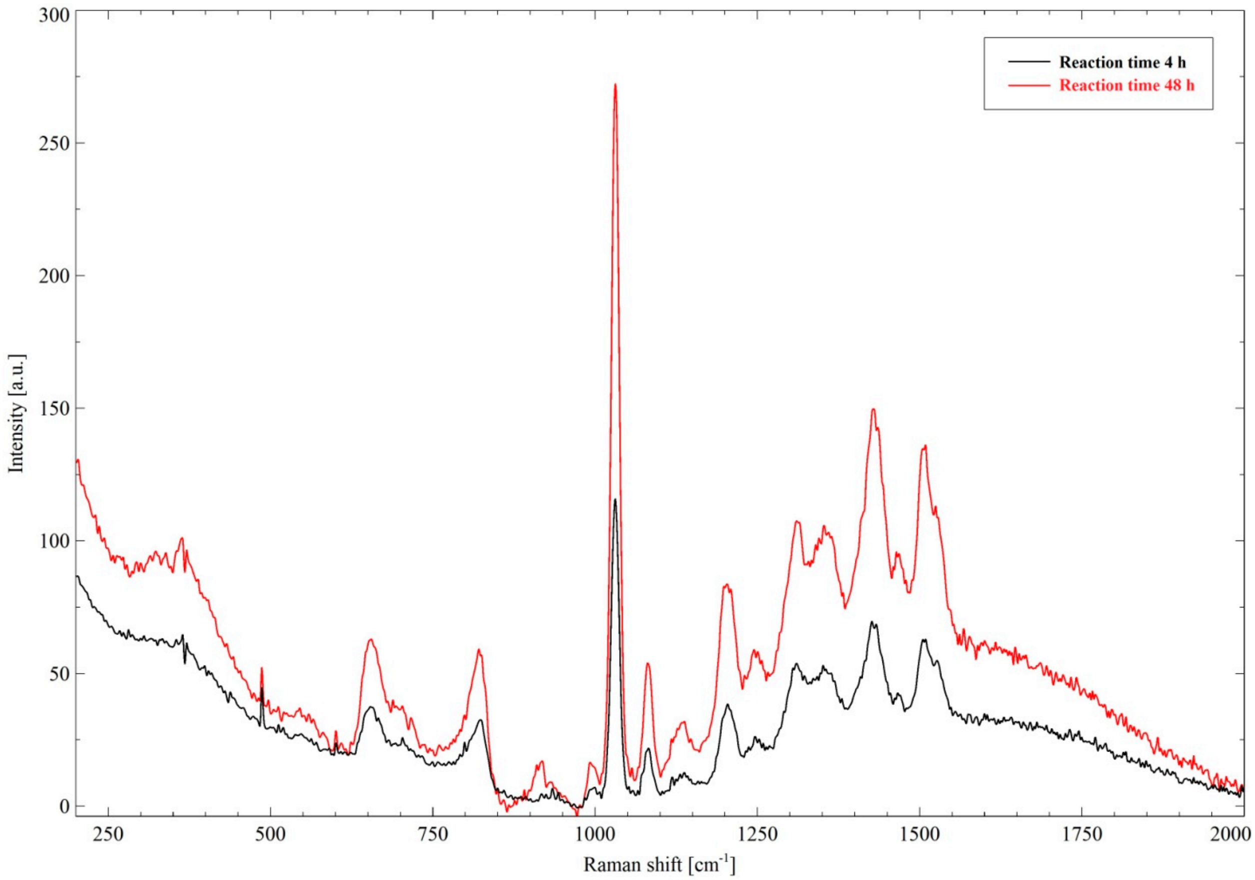

Figure S5. Raman spectra of compound **2** after reaction times of 4 h and 48 h.

Table S1. Results of density measurements of PMT obtained by a nitrogen pycnometer.

n	density [g/cm^3]
**1**	1.543738
**2**	1.548451
**3**	1.551488
**4**	1.557145
**5**	1.554724
**6**	1.556915
**7**	1.555381
**8**	1.560757
**9**	1.556692
**10**	1.558824
**11**	1.558159
**12**	1.560052
**13**	1.565157
**14**	1.564528
**15**	1.560474
**Average**	1.556832
**St. dev.**	0.005671

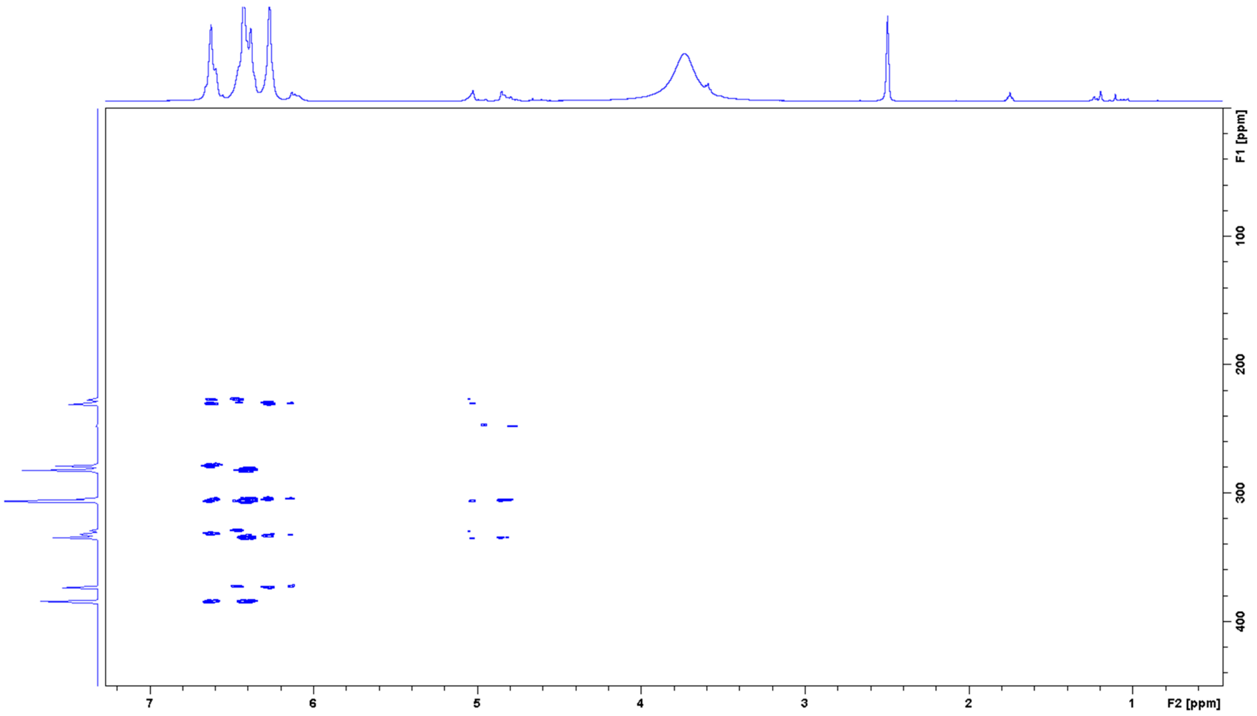

Figure S6. ^{1}H-^{15}N NMR spectrum of compound **2**.

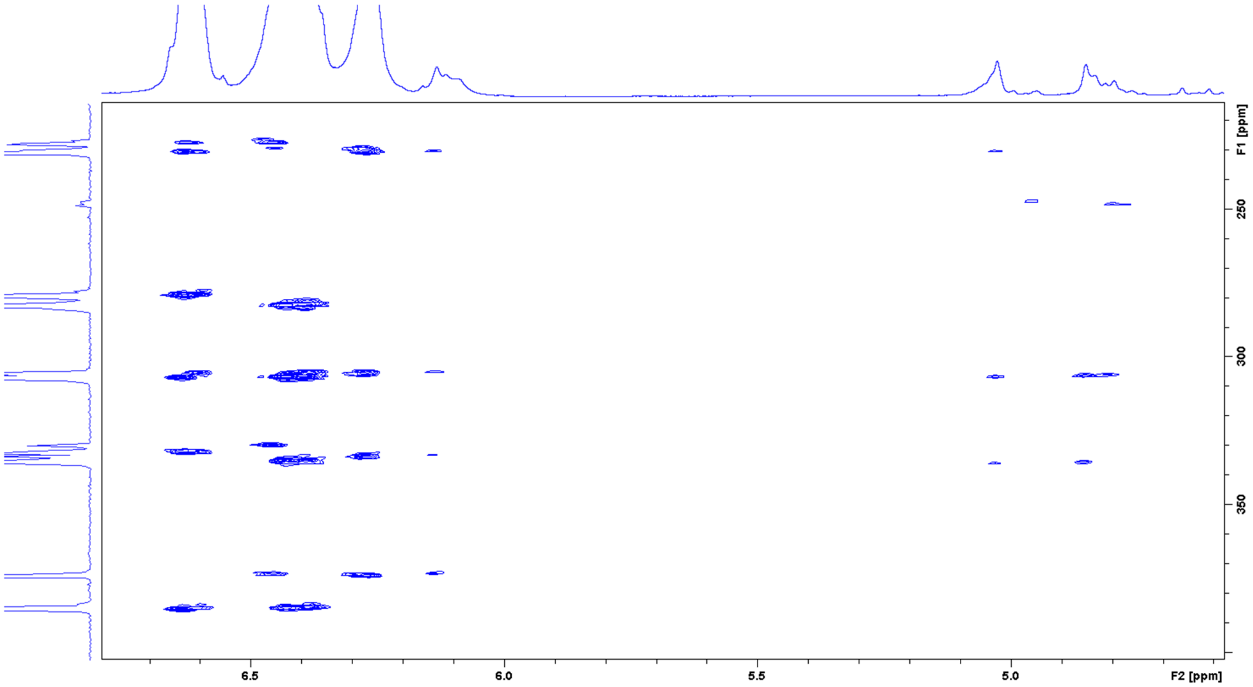

Figure S7. ^{1}H-^{15}N NMR spectrum of compound **2** (zoomed in).
